# Methodology for mapping reviews, evidence maps, and gap maps

**DOI:** 10.1017/rsm.2025.25

**Published:** 2025-06-16

**Authors:** Hanan Khalil, Vivian Welch, Matthew Grainger, Fiona Campbell

**Affiliations:** 1 Department of Public Health, School of Psychology and Public Health, La Trobe University, Melbourne, VIC, Australia; 2 Bruyere Research Institute, Ottawa, ON, Canada; 3 School of Epidemiology and Public Health, University of Ottawa, Ottawa, ON, Canada; 4 Norwegian Institute for Nature Research, Trondheim, Norway; 5 Population Health Sciences Institute, Newcastle University, Newcastle, UK

**Keywords:** mapping reviews, evidence synthesis, evidence maps, gap maps

## Abstract

Mapping reviews are valuable tools for synthesizing and visualizing research evidence, providing a comprehensive overview of studies within a specific field. Their visual approach enhances accessibility, enabling researchers, policymakers, and practitioners to efficiently identify key findings, trends, and knowledge gaps. These reviews are particularly significant in guiding future research, informing funding decisions, and shaping evidence-based policymaking. In environmental science—similar to health and social sciences—mapping reviews play a crucial role in identifying effective conservation strategies, tracking interventions, and supporting targeted programs.

Unlike systematic reviews, which assess intervention effectiveness, mapping reviews focus on broad research questions, aiming to chart the existing evidence on a given topic. They use structured methodologies to identify patterns, gaps, and trends, often employing visual tools to enhance data accessibility. A well-defined scope, guided by inclusion and exclusion criteria, ensures a transparent study selection process. Comprehensive search strategies, often spanning multiple databases, maximize evidence capture. Effective screening, combining automated and manual processes, ensures relevance, while data extraction emphasizes high-level categories such as study design and population demographics. Advanced software tools, including EPPI-Reviewer and MindMeister, support data extraction and visualization, with evidence gap maps highlighting robust areas and research voids.

Despite their advantages, mapping reviews present challenges. The categorization and coding of studies can introduce subjective biases, and the process demands substantial resources. Automation and artificial intelligence offer promising solutions, improving efficiency while addressing integration and multilingual limitations. As methodological advancements continue, interdisciplinary collaboration will be essential to fully realize the potential of mapping reviews across scientific disciplines.

## Highlights

### What is already known

- Mapping reviews provide a structured approach to synthesizing large bodies of research and are widely used in health and social sciences.
- These reviews visually organize information, improving accessibility and aiding decision-making.
- Evidence gap maps support research prioritization by identifying well-studied areas and knowledge gaps.

### What is new

- The role of mapping reviews in environmental science is increasingly recognized for tracking conservation strategies and informing policies.
- Automation, artificial intelligence, and specialized software tools are enhancing the efficiency of mapping reviews.
- Methodological refinements are addressing challenges such as subjectivity in coding and multilingual limitations.

### Potential impact for RSM readers

- Provides insights into how mapping reviews can be leveraged across various scientific disciplines, including environmental research.
- Highlights advancements in technology and automation that improve the accuracy and efficiency of mapping reviews.
- Offers a framework for integrating mapping reviews into evidence-based policymaking and prioritizing future research.

## 1 Introduction

Mapping reviews and evidence gap maps (EGMs) are used in research synthesis and evidence-based decision-making to navigate complex bodies of knowledge and identify key insights. Mapping reviews and EGMs involve systematically categorizing and organizing existing literature or data sources, enabling researchers and decision-makers to conceptualize the landscape of a field or topic, identifying trends, gaps, and areas of consensus or contention.

Mapping reviews and evidence gap maps (EGMs) are not only tools for organizing large bodies of literature—they are increasingly central to structured evidence-informed decision-making (EIDM) processes.^1^ By systematically identifying where evidence is robust and where gaps persist, these tools can guide priority setting, policy formulation, and resource allocation in real-world contexts. For example, policymakers may use an EGM to determine which environmental interventions have sufficient evidence to justify implementation, or where further primary research is needed before action. Mapping reviews can also be integrated into formal EIDM frameworks such as those promoted by the WHO or GRADE Evidence to Decision (EtD) frameworks, ensuring that decisions are grounded in a comprehensive and unbiased understanding of the evidence landscape. Strengthening the link between mapping outputs and policy uptake requires active engagement with stakeholders during review planning and dissemination stages, to ensure that findings are aligned with decision-makers’ needs and timelines.^2^

By synthesizing identified evidence using a coherent framework, mapping reviews enable stakeholders to gain a holistic understanding of the subject matter and make informed decisions based on a comprehensive evidence base.^3^

Evidence gap maps, on the other hand, are graphical representations of the relationships and interactions among various components within a system. In the context of research synthesis and evidence-based decision-making,^4^ evidence gap maps can depict the complex interplay of factors influencing a particular issue or problem. By visualizing the connections between different variables or factors, evidence gap maps help researchers and decision-makers identify leverage points, unintended consequences, and potential interventions for addressing the issue at hand.^5^ Together, mapping reviews and evidence gap maps provide valuable tools for synthesizing and interpreting complex information, identifying research gaps, and guiding evidence-based decision-making processes across a wide range of disciplines and domains.

Mapping reviews appeared in the literature in 2007, with an increase in their numbers over the years.^6^ This led to several organizations developing methods for their conduct and reporting.^7^ For example, the EPPI-Centre, Social Care Institute for Excellence (SCIE), Collaboration for Environmental Evidence (CEE), the international initiative for impact evaluation (3ie) and Campbell Collaboration have all developed different variants of mapping reviews to address the need to map the increasing volume of literature in a particular topic in a meaningful way for researchers and policy makers. However, this created a number of terminologies describing the same methodology, including evidence maps and gap maps. Examples of these included system maps, systematic maps, systematic mapping, and mapping reviews. James et al. and Haddaway et al. detailed a methodology for systematic mapping for environmental science with a step-by-step approach on how to conduct them.^8^ ^,^ ^9^ This has led to other researchers taking an interest in developing the methodology using robust frameworks.^8^ ^,^ ^10^ Some of these methodologies are less developed than others regarding the clarity of each step of the methodology and what needs to be considered to undertake a mapping review.

Campbell et al. recognized the increasing confusion in the terminology and detailed the differences in terminology between the terms used.^1^ The authors first clarified the differences between scoping reviews and mapping reviews, specifically in terms of the depth of data extracted. Scoping reviews and mapping reviews both tend to address broad research questions, but the level of data extraction varies between the two methods. Scoping reviews tend to include in-depth data extraction, including findings, summaries, recommendations, and areas for further research.^1^ ^,^ ^3^ Mapping reviews, on the other hand, tend to include a high level of data extraction, which is mostly categorized according to a framework. Khalil and Tricco 2023 progressed this work further and clarified the differences between mapping reviews and EGMs. They stated that mapping reviews may or may not include evidence gap maps. They clarified that evidence gap maps (EGMs) can be used as a tool to visualize the data in mapping reviews. This is consistent with the latest work published by Khalil et al., who found that 48% of all mapping reviews included an EGM, while the remainder of reviews included other visuals.^6^ EGMs may also exist as a standalone evidence synthesis output and not be part of a mapping review.^1^ ^,^ ^3^

The methodological framework for this present study is guided by the latest work published on mapping reviews and EGMs, aiming to provide up-to-date guidance for environmental scientists seeking to map the evidence addressing a broad research question. To enhance clarity around how key findings were derived, we followed a structured, multi-step methodological process that built on recent advances in mapping review methodology, including guidance from Khalil et al. and Li et al.^2^ ^,^ ^6^ We began by systematically identifying and analyzing existing methodological and guidance documents related to mapping reviews and evidence gap maps (EGMs), using a scoping review approach guided by the Joanna Briggs Institute (JBI) framework and PRISMA-ScR. We extracted data on conceptual definitions, methodological characteristics, and reporting practices from included sources and synthesized these using both descriptive statistics and thematic analysis. From this synthesis, we identified common methodological elements across mapping reviews, which were then consolidated into a draft conceptual framework (Table 1). This framework was developed iteratively by categorizing and clustering steps frequently reported in methodological and guidance documents and validated through team consensus to ensure it reflected both diversity and consistency across the field.^1^ ^–^ ^3^ ^,^ ^6^

**Table 1:** Outlines the steps of undertaking a mapping review, with examples from environmental science research

Step	Description	Example from environmental science research
1. Define the review scope	Determine the research question, objectives, and scope, including the population, intervention/exposure, comparators, and outcomes (PICO/PECO).	*Example:* Assessing the impacts of climate change on freshwater biodiversity. *PICO* –*Population:* Freshwater ecosystems, *Intervention*: Climate change effects. *Outcome:* Biodiversity changes.
2. Develop a protocol	Create a detailed protocol that outlines the review methodology, including search strategy, inclusion/exclusion criteria, and data extraction methods.	*Example:* Protocol to identify studies assessing the impact of climate change on freshwater species richness and distribution.
3. Searching the literature	Perform a comprehensive literature search across multiple databases and sources to identify relevant studies.	*Example:* Searching databases like Web of Science, Scopus, and Google Scholar using keywords like “climate change,” “freshwater biodiversity,” and “species richness.”
4. Screen studies	Screen the identified studies for relevance using predefined inclusion and exclusion criteria.	*Example:* Screening studies that specifically focus on empirical research related to climate change impacts on freshwater ecosystems, excluding studies on marine ecosystems.
5. Data extraction	Extract key data from the included studies, such as study design, geographic location, types of ecosystems, and outcomes measured.	*Example:* Extracting data on the geographic regions studied, the types of freshwater species affected, and the observed changes in biodiversity.
6. Data coding and categorization	Code and categorize the extracted data based on predefined themes, categories, or a framework.	*Example:* Categorizing studies based on regions (e.g., temperate vs. tropical), types of climate change impacts (e.g., temperature rise and altered precipitation), and types of freshwater species (e.g., fish and invertebrates).
7. Risk of bias assessment (optional)	This step is useful if the mapping review explicitly examines outcomes, effect sizes, or intervention impacts.	*Example:* assessing studies investigating the impact of high temperature on forests, plants, and animals.
8. Utilize visual presentation of the data to facilitate analysis and usability of the review (could include an EGM)	Visualize the data using charts, graphs, or tables to summarize the findings and highlight research gaps.	*Example:* Creating a heat map showing the geographic distribution of studies on climate change impacts on freshwater biodiversity, identifying regions with limited research.
9. Interpret and discuss findings	Analyze the results to identify trends, patterns, and gaps in the literature, and discuss the implications for future research and policy.	*Example:* Interpreting the map to identify that most studies focus on temperate regions, with limited research on tropical freshwater ecosystems, suggesting a need for more studies in underrepresented areas.
10. Report the findings	Write a comprehensive report or publication summarizing the review process, results, and conclusions.	*Example:* Publishing a mapping review report in a journal like *Environmental Research Letters*, Campbell Collaboration, highlighting key research gaps and recommendations for future studies.

The steps for undertaking a mapping review are presented in Table 1.

## 2 Methodology

### 2.1 Defining the scope

Mapping reviews differ from systematic reviews by addressing a broad question and mapping the evidence related to a topic. Examples of these research questions include the extent of evidence on air pollution and health, types of outcomes and populations studied, the ethics of artificial intelligence in healthcare, and mapping of protests against fossil fuel and low-carbon energy projects.^11^ ^–^ ^13^ The topic in question is broad and does not lend itself to a systematic review of effectiveness, feasibility, appropriateness, or meaningfulness. Hence, a mapping review methodology seems to be the most appropriate methodology to follow in this case. Other examples of research questions for mapping reviews include: “what evidence exists concerning…?”, “how much research is available regarding…?”, and “what is the current state of knowledge about…?”.^8^

The mapping review question guides the development of the inclusion and exclusion criteria for undertaking the review. Mapping reviews could address any form of research question, bibliometric analysis of a topic, exploratory topics, associations, and methodological analysis. Other research questions, such as prevalence, prognostic, and diagnostic test accuracy, have also been used.^6^ Inclusion criteria should align with the purpose of the review and define the parameters of the populations, interventions/concepts, outcomes, and settings of the review question. The types of evidence sources need to be identified clearly in this section, as it will direct the breadth and depth of the included studies. An example of a mapping review published in environmental science is the one by Dominski et al., where they analyzed the state of the art on the effects of air pollution on human health through a mapping review of existing systematic reviews and meta-analyses.^14^

While mapping reviews are often conducted in areas with large volumes of literature, volume alone does not justify the choice of a mapping methodology. A mapping review is most appropriate when the research question is broad, exploratory, and aims to capture the landscape of evidence rather than test specific hypotheses.

It is recommended to develop a pre-planned protocol for the mapping review to reduce the risk of reviewer bias, ensure transparency, and reduce research waste by avoiding duplication.^15^ Developing a priori protocols ensures a high-quality review and adherence to a well-defined question and setting the scope for the review.^15^ Our earlier work on mapping reviews highlighted that more than 70% of them did not include a protocol and only a small percentage were registered in Open Science Framework (https://osf.io/), International prospective register of systematic reviews PROSPERO https://www.crd.york.ac.uk/prospero/) or the International Platform of Registered Systematic Review and Meta-analysis Protocols website; (https://inplasy.com/).^6^ Currently, PROSPERO does not register scoping reviews, possibly due to the lack of critical appraisal of included studies. Since both mapping and scoping reviews are closely related, particularly in terms of having an optional step of critical appraisal, they are unlikely to be accepted in PROSPERO, and therefore, we recommend their registrations in either Open Science Framework or Figshare (https://figshare.com/), a repository where users can make all of their research outputs citable or in journals which accept protocols, such as Campbell, Joanna Briggs Institute Evidence Synthesis, and Environmental Evidence.

### 2.2 Search strategy

Mapping reviews typically include a variety of studies with different designs to capture the required information. When developing a search strategy for large reviews, it is important to run a preliminary search on two or more databases to determine the magnitude of the evidence in the area. Mapping reviews typically involve between one and 246 sources of information; therefore, it is important to plan the best way of undertaking searches amongst the reviews team members.^6^ Searching for systematic reviews require a thorough and reproducible search of a variety of sources, such as Content—Environmental Science Collection—LibGuides at ProQuest to capture relevant studies. Searching one database, such as Web of Science, is inadequate, as there are numerous specialized databases for a range of disciplines. Consulting with a library specialist is advisable to identify the relevant search terms and databases for the topic of interest.^16^

There are several guidelines for searching databases in the literature, the best search strategy usually strikes a good balance between specificity and sensitivity.^17^ ^–^ ^19^ Bramer et al. have identified a 15-step methodology for planning and creating a multi-database search strategy. The first nine steps are standard to other search guidelines, which include formulating a clear question, identifying articles that can answer the research question, and developing key concepts to address the various elements of the research question.^17^ The six subsequent steps focus on creating the appropriate syntax for each database used, as they are different, for example, using parentheses, Boolean operators, and truncation. Follow-up steps include optimizing the search by identifying extra terms and adding them to the test strategy. Evaluating the initial results, checking for errors, translating to other databases, and testing the search strategy are the final stages of the methodology and are essential to retrieve all articles.^17^

Aromataris et al. developed a three-step search strategy for reviews, which could also be used. This includes developing a search strategy in one database to identify keywords and relevant search terms.^18^ ^,^ ^19^ The second step involves entering these keywords in other databases using the relevant syntaxes for each database. The third and final step includes checking the reference list of the included studies to capture as many relevant studies as possible.^18^ Irrespective of which method chosen, determining the acceptable number of results for screening typically involves negotiation with the review team.

There are several strategies that can be used to minimize bias in searching; these include avoiding using additional terms that can restrict searches. For example, if the research question is about strategies to reduce the risks of air pollution, it is best to avoid words such as “reducing,” “prolonging,” etc., as they result in restrict the number of articles retrieved. Striking a balance between specific and general terms versus important and unimportant is crucial to produce a sensitive and specific search. Another strategy to minimize bias in searching is to avoid overlapping terms, for example: ecosystem and habitat, climate change and global warming, and conservation and preservation.

### 2.3 Screening

Screening studies for a mapping review involve a systematic process to identify relevant literature based on pre-defined inclusion and exclusion criteria. Initially, a comprehensive search is conducted across multiple databases, ensuring that all potentially relevant studies are captured. The titles and abstracts of the retrieved articles are then screened to remove irrelevant studies, duplicates, and those that clearly do not meet the inclusion criteria.^1^ This step is typically done by two or more reviewers independently to minimize bias. Studies that pass this initial screening are subjected to full-text review, where the content is thoroughly examined to determine their eligibility for inclusion in the mapping review. Throughout the process, a record of decisions and justifications for inclusion or exclusion is maintained to ensure transparency and replicability. This methodical screening helps ensure that the final set of studies accurately reflects the breadth of research relevant to the topic being mapped.^6^

### 2.4 Data extraction

The process of data extraction for mapping reviews can be challenging for many authors due to multiple factors, such as the plethora of literature included, the lack of guidance, and inconsistencies that exist in data extraction, and the type of evidence included in the type of reviews. It is recommended that data extraction for these large reviews be restricted to descriptive meta-data or high-level categories (such as study design used or population), since the objective of these reviews is to identify and describe the existing evidence rather than extract detailed findings.^8^

Since data extraction is not as exhaustive as in systematic reviews, it is important that the extracted data address the research questions posed in the review. Examples of data extraction include demographics, types of interventions, outcome measures collected, types of interventions, etc. These data can also be represented using descriptive analysis such as pivot tables, histograms, pie charts, and others.^20^

Another method employed by Fiest et al. is a coding technique using NVivo 11 Pro (QSR International), where multiple rounds of coding were used to make sense of the literature.^10^ Codes were then inductively developed and defined. This process guided the second round of coding, in which text passages were accordingly coded.^10^ ^,^ ^21^ ^,^ ^22^ This technique has been very useful when exploring challenging questions addressing relationships between variables. For example, exploring links between collaborative conservation processes and outcomes, where the mechanisms of change may not be well understood.

### 2.5 Data coding and characterization

A mapping framework is a structured approach for organizing and visualizing data to provide a comprehensive overview of complex information, as it allows for the clear presentation of relationships, processes, and systems. By visually mapping data, researchers and practitioners can identify patterns, gaps, and connections that might not be immediately evident through textual analysis alone. This enhanced understanding facilitates more informed decision-making and effective communication of findings to diverse audiences.^5^ ^,^ ^23^

Unlike bibliometric analyses that primarily quantify publication trends and metadata, coding and categorization in mapping reviews involve thematic analysis aligned with the review framework. This process allows researchers to extract conceptual patterns, relationships, and thematic distributions that inform a deeper understanding of content, not just bibliometric attributes.

There are several types of mapping frameworks, each suited to different research questions and objectives.^7^ Conceptual frameworks, for instance, help in illustrating theoretical relationships between concepts and variables, making them ideal for exploratory studies or theoretical research. Logic models, on the other hand, are more pragmatic and focus on depicting the sequence of actions and outcomes, thus proving useful in program evaluation and implementation research. Other frameworks like flowcharts, mind maps, and systems maps serve distinct purposes, from detailing procedural steps to illustrating complex interdependencies within a system. The choice of framework depends largely on the specific needs of the research, such as whether the aim is to explore theoretical constructs or to track the practical implementation of a program.

Developing a mapping framework tailored to research objectives involves several critical steps. First, clearly define the research question and objectives to determine the type of data and relationships that need to be visualized. Next, select the most appropriate framework type based on the nature of the research and the audience for the findings. Gather and organize the necessary data, ensuring that it is both comprehensive and accurate. Then, construct the framework, starting with the primary components and gradually adding detail to illustrate relationships and processes effectively. This can be an iterative process whereby continuous consultation with researchers and stakeholders may be necessary to address the research question.^23^ Throughout this process, it is important to remain flexible and open to refining the framework as new insights emerge.^23^ ^,^ ^24^ Finally, validate the framework through peer review or stakeholder feedback to ensure it accurately represents the intended information and serves its purpose effectively.^25^

### 2.6 Risk of bias assessment

Risk of bias (ROB) or quality assessment is not always necessary in mapping reviews, particularly when the objective is to map the breadth of existing evidence without assessing effectiveness or outcomes. However, if the research question explicitly examines outcomes, effect sizes, or intervention impacts, then incorporating a quality assessment becomes essential. Mapping reviews addressing such analytical questions should incorporate tools suitable for assessing methodological rigor, while broader scoping-focused maps may not require this step.

### 2.7 Creating evidence gap maps

Creating evidence gap maps involves a systematic process based on a well-defined mapping framework.^7^ ^,^ ^26^ Once the relevant studies are collected, they are categorized based on predefined criteria, such as study design, outcomes measured, and population characteristics. The categorized data is then plotted onto the framework, highlighting areas with robust evidence and those with significant gaps. This visual representation helps identify where more research is needed, guiding future studies and funding priorities.^1^ ^,^ ^26^

Techniques for representing relationships, connections, and pathways in evidence gap maps are crucial for effectively conveying the complexity of the evidence landscape.^23^ One common technique is the use of nodes and links, where nodes represent individual studies or pieces of evidence, and links illustrate the relationships between them. Color coding and varying the size of nodes can indicate the strength or quality of evidence, while directional arrows can show causality or influence pathways.^7^ Additionally, clustering related nodes together can help visualize thematic areas within the evidence base. These visual cues make it easier to interpret the map and to quickly identify key findings and gaps.^1^

Several software tools and platforms are available for creating mapping reviews and EGMs. For example, EPPI-Mapper can generate EGMs from JSON files, which can be created in EPPI-Reviewer. (Eppi-Mapper 2023; Eppi-Reviewer, 2022; and EviAtlas) EPPI-Reviewer offers robust functionalities for data extraction, coding, and visualization. EPPI-Reviewer can also support the entire review process, including screening and managing studies. It facilitates teamwork and enables reviewers to undertake double screening and coding, blinded to the decisions of their co-reviewer. EviAtlas is an open-access software tool for producing interactive, attractive tables and figures that summarize the evidence base.^27^

Mind mapping software, such as MindMeister and XMind, can also be adapted for evidence mapping, offering intuitive interfaces for creating and sharing visual maps.^28^ UCINET and Netdraw (Analytic Technologies), a combined software package for network analysis and visualization, were also used to further analyze the relationships between variables in mapping reviews.^7^ These tools facilitate the efficient creation of evidence gap maps, allowing researchers to focus on analyzing and interpreting the evidence rather than getting bogged down in technical details.

## 3 Discussion

Mapping reviews have several strengths that make them valuable tools in synthesizing and visualizing research evidence. One of their primary strengths is their ability to provide a comprehensive overview of existing research in a particular field, making it easier to identify patterns, trends, and gaps.^1^ ^,^ ^6^ By visually organizing complex information, mapping reviews enhance the accessibility and interpretability of data for diverse audiences, including researchers, policymakers, and practitioners. Additionally, they facilitate the identification of areas where evidence is robust and where further investigation is needed, guiding future research efforts and funding decisions. The visual nature of mapping reviews also aids in effective communication, enabling stakeholders to identify key findings and their implications.^3^

The potential applications of mapping reviews and system maps are diverse. In research, they can serve as foundational tools for conducting systematic reviews, meta-analyses, and identifying research gaps. Policymakers can use results from mapping reviews to identify gaps in the literature. This can then inform evidence-based decision-making, ensuring that policies are grounded in the most comprehensive and current data available.^1^ ^,^ ^6^

In environmental science practice, mapping reviews are helpful in guiding the implementation of best practices and interventions by highlighting effective strategies and locating areas that require further consideration. These reviews can, for example, track the effectiveness of conservation initiatives across different ecosystems and geographical regions, enabling the design of targeted and impactful environmental programs.^29^ By visually arranging existing research, mapping reviews help practitioners identify which strategies have been successful and under what conditions, thereby informing evidence-based decision-making and policy development. This approach ensures that interventions are not only scientifically sound but also contextually relevant, ultimately leading to more sustainable and effective environmental management practices.^30^

However, mapping reviews also have limitations that must be considered. The process of categorizing and coding studies can introduce subjective biases, potentially affecting the accuracy and objectivity of the map.^10^ ^,^ ^21^ ^,^ ^22^ Additionally, creating and maintaining up-to-date maps can be resource-intensive, requiring significant time and expertise.

The International Collaboration for Automation of Systematic Reviews (ICASR) has proposed principles for using automation in systematic reviews, emphasizing its potential to enhance the quality and efficiency of these reviews. However, scoping reviews and mapping reviews, which address broader research questions and use diverse evidence sources, require specific guidance when using automation tools. A recently published paper introduced several reliable automation tools that can support large reviews, marking a significant advancement in the field.^31^

Although there is currently no dedicated reporting guideline exclusively for mapping reviews, the PRISMA-ScR (Preferred Reporting Items for Systematic reviews and Meta-Analyses extension for Scoping Reviews) provides a practical and structured framework that can be adapted to guide the reporting of mapping reviews. PRISMA-ScR emphasizes transparent and comprehensive reporting of the review rationale, objectives, eligibility criteria, sources of evidence, and data charting processes—all of which align closely with the methodological steps undertaken in mapping reviews. While mapping reviews may differ from scoping reviews in terms of data extraction depth and visualization methods, the PRISMA-ScR checklist remains highly applicable in ensuring methodological transparency and reproducibility. Moreover, ROSES (Reporting standards for Systematic Evidence Syntheses), a pro forma and flow diagram designed specifically for systematic reviews and systematic maps in the field of conservation and environmental management.^32^ Until a tailored guideline is developed for mapping reviews, adapting PRISMA-ScR and ROSES supports best practices and enhances the credibility, usability, and dissemination of findings in evidence mapping exercises.^33^ Our team is currently working on dedicated reporting guidelines for mapping reviews.

While automation for both systematic and scoping reviews is evolving, this guidance highlights the integration of automation tools with human expertise to improve review processes. However, key limitations remain, including the lack of multilingual availability, difficulty in comparing tools, and limited tool integration, which complicates the review process. Despite these challenges, the guidance offers valuable support for researchers using automation in scoping reviews.

There are several areas where further research and methodological refinement are needed. One area for improvement is the need for advanced analytical techniques and software tools that can handle the increasing complexity and volume of research data.^6^ Innovations in artificial intelligence and machine learning hold promise for automating parts of the mapping process, thereby increasing efficiency and reducing potential biases. Further research is also needed to explore the integration of qualitative data into mapping reviews, providing a more holistic understanding of research landscapes. As these methodological advancements are made, mapping reviews will become even more powerful tools for synthesizing and applying research evidence across various domains.

## 4 Conclusion

Mapping reviews for environmental scientists reveal key findings that illustrate the breadth of research across various environmental issues, such as climate change, biodiversity conservation, and pollution control. These reviews highlight areas of strong evidence, such as the efficacy of certain conservation strategies or the impacts of pollutants on ecosystems, while also identifying significant knowledge gaps that need further exploration. The importance of methodological rigor in these reviews and system maps cannot be overstated, as it ensures the accuracy, reliability, and transparency of the findings, ultimately guiding effective policy and practice. For researchers and practitioners interested in using this methodology, it is recommended to adhere to standardized protocols, use advanced analytical tools to manage complex data, and remain vigilant about potential biases in data collection and analysis. Engaging in continuous methodological refinement and seeking interdisciplinary collaboration can further enhance the utility and impact of mapping reviews in the environmental sciences.

## Data Availability

All data are available from the references list.
